# A critical stress model for cell motility

**DOI:** 10.1186/1742-4682-9-49

**Published:** 2012-11-24

**Authors:** Mehrnush Mehrayin, Farhad Farmanzad, Masoud Mozafari, Daryoosh Vashaee, Lobat Tayebi

**Affiliations:** 1 Department of Mechanical Engineering, Iran University of Science and Technology, Tehran, Iran; 2 Helmerich Advanced Technology Research Center, School of Material Science and Engineering, Oklahoma State University, Tulsa, OK 74106, USA; 3 Helmerich Advanced Technology Research Center, School of Electrical and Computer Engineering, Oklahoma State University, Tulsa, OK 74106, USA; 4 School of Chemical Engineering, Oklahoma State University, Stillwater, OK 74078, USA

**Keywords:** Finite difference, Cell motility, Continuum model, Critical stress

## Abstract

A detailed theoretical model that combines the conventional viscoelastic continuum description of cell motion with a dynamic active stress is presented. The model describes the ameboid cells movement comprising of protrusion and adhesion of the front edge followed by detachment and movement of the tail. Unlike the previous viscoelastic descriptions in which the cell movement is steady, the presented model describes the “walking” of the cell in response to specific active stress components acting separately on the front and rear of the cell. In this locomotive model first the tail of the cell is attached to the substrate and active stress is applied to the front of the cell. Consequently, the stress in the tail increases. When the stress in the tail exceeds a critical value, namely critical stress, the conditions are updated so that the front is fixed and the tail of the cell is detached from the substrate and moves towards the front. Consequently, the stress in the tail decreases. When the stress goes to zero, the starting conditions become active and the process continues. At start the cell is stretched and its length is increased as the front of cell migrates more than the rear. However, after several steps the front and rear move equally and the cell length stays constant during the movement. In this manuscript we analyzed such cell dynamics including the length variation and moving velocity. Finally, by considering this fact that at the single-cell level, interactions with the extracellular environment occur on a nanometer length scale, the value of critical stress was estimated.

## Introduction

Cell motility is based on different biological events and pathological processes. In this regard, understanding the forces between the cells and substrates responsible for cell motility not only allows the underlying of many pathological processes but also holds promise for designing novel engineered materials for tissue engineering and regenerative medicine [1-3]. Migration involves different coordinated events such as protrusion of pseudopodia, formation of new adhesions, maturation of traction, and release of old adhesions [4]. To obtain suitable physiological effects, cell motility must maintain a specific speed and direction in response to environment stimuli. As a challenging issue, migration control by gradients of dissolved and surface-attached chemicals has been investigated for decades [5-8]. The motility of different cells involves some stages. According to Mitchison and Cramer [9] the motility of ameboid cells includes four different steps of protrusion, attachment to substrate, translocation of cell body, and detachment of its rear. Cells first extend localized protrusions at the leading edge, which take the form of lamellipodia, filopodia or pseudopodia. Most current models explain force generation at the leading edge by localized actin polymerization and crosslinking (or gelation) of actin filaments. In the second step, the protrusion anchors to other cells or to the substrate [10]. A protrusion maintains its stability by the formation of new adhesive complexes which act as sites for molecular signaling as well as transmitting mechanical force to the substrate. In the next step, actomyosin filaments pull the cell toward the protrusion in fibroblasts by a contract at the cell front, whereas in other kind of cells, contraction is at the rear and the cytoplasm is compressed from the front. Finally, in the last step, the cell disconnects the adhesive contact, which allows the tail of the cell to follow the main body [11,12].

During the last few decades, numerous models of cell motility have been reported. In 1989, Lauffenburger [13] studied the correlation between cell speed and receptor density and affinity. He also reported a model in one-dimension and explained three regions as lamellipod, cell body, and uropod. In 1991, DiMilla et al. [14] analyzed the interactions of the cell and the substrate by additional Maxwell elements at the front and the rear. In their model the cells consisted of discrete subunits, each with a spring, dash-pot and contractile element connected to each other in parallel. They showed that this bell-shaped distribution of the cells speed could be described by an asymmetry in adhesiveness from preferable binding at the cell front.

Recently, a method has been studied and applied to a two-dimensional model of nematode sperm by Bottino et al. [15]. They modeled the interactions of the cell and the substrate by a viscous drag between the substrate and the cell. They also modeled the polymerization of actin network at the forward edge and its disassembly at the rear of the cell both for single and interacting cells. This model was biochemically regulated and described the fixed continuous movements of the cell.

These models usually treat the cell body as a combination of dashpots and springs, and solve the resulting force balance equations at each node. Although this approach gives qualitative perceptions into the features of the cell motility, the cell body is more accurately described as a possibly multi-phase continuum. Therefore, it seems that modeling of the cell by means of continuum approach would be more appropriate.

More recently Gracheva and Othmer [16] developed a continuum model for the cell as a viscoelastic material. They studied the spatial variability of elasticity and viscosity coefficients in addition to the gradient in physical characteristics of the substrate. This approach gave them the opportunity of modeling different kinds of cells. In 2010, Sarvestani [17] described a physical model to study the motility of a contractile cell on a substrate. The model demonstrated that the motility of cells significantly depended on the rigidity of the substrate. This dependency was rooted in the regulation of actomyosin contractile forces by substrate at different anchorage points. It suggested that on stiffer substrates, the traction forces required for cell translocation acquire larger magnitude. However, this results in weaker asymmetry which causes slower cell motility. Also, on soft substrates, the model suggested a meaningful relationship between the rigidity of the substrate and the speed of cell movement.

As we explained earlier, the motility of ameboid cells includes four steps of protrusion, adhesion to substrate, cell body movement and detachment of cell tail. In the previous studies, these steps have not been considered for the cell motility modeling. Instead, a steady movement was attributed to the cell. Although the previous steady models were in agreement with the experimental data in term of the length and the position of the cell, in order to study the stress generated in the cell during its motion a model based on the steps of ameboid cell motility, which is closer to the motion of a real cell, is necessary.

**Figure 1:**
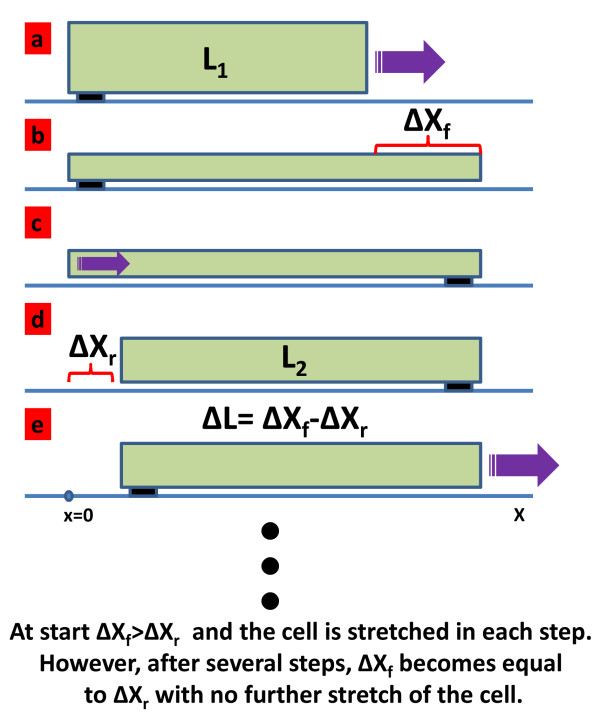
**The steps of cell motility we considered in our model.** (**a**) Cell rear is adhered to the substrate and the cell front is moving. (**b**) The length of the cell is increased toL_1_+ Δx_f_. (**c**) After the stress is reached to a certain degree (critical stress value) the rear of the cell detached and start moving forward while the front of the top is adhered to the substrate. (**d**) The rear of the cell moves as much as Δx_r_. (**e**) The process repeat with the new cell length which is equal to L_1_+ΔL where ΔL=Δx_f_–Δx_r_. Note that the length of the cell cannot stretch indefinitely and it will reach to a point that the rear and front of the cell have same displacement.

In this study, we present a critical stress two-step walking model for the cell motility, which is of great interest to scientists dealing with tissue engineering and nanomedicine. The boundary conditions in our model are closer to the actual motion of the cell [18-20] which can be schematically shown as in Figure 1. As it is seen in this figure, the cell front moves while the rear is attached to the substrate. When the stress in the front of the cell exceeds a critical value, the front stops and the rear side starts migrating. At the beginning of the process, the front moves longer distance than the rear at each step resulting in a stretch in the length of the cell. However, after several steps the stretch counter acts the front motion so that the front and the rear move equal distances at each step. Therefore, the length of the cell reaches an equilibrium value. The equilibrium length depends on the cell properties.

## Equations of motion and boundary conditions

Generally, investigating cell functions such as migration and adhesion as well as differentiation requires accurate mimicking of the in vivo microenvironment. This mimicking of the natural extra cellular matrix requires biomaterials that are tunable down to the nanometer length scale. In this work, a one dimensional simulation used for cell motility is based on the classical continuum model for ameboid cell. This model was previously developed by Gracheva et al. in 2004 [16]. We extended the model with applying variable boundary conditions to explain the cell motility in a critical stress two-step walking model. In brief, following ref. [16], the equation of motion for the cell is defined as:

(1) $$\frac{\partial \sigma }{\partial x}=\beta \left(x\right)\frac{\partial u}{\partial t}$$

In which *x* is position, *t* is time, *u* is the displacement of the cell, σ is the stress along the cell, and β is an effective drag coefficient or friction. σ is governed by the following equation:

(2) $$\sigma =E\left(x\right)\frac{\partial u}{\partial x}+\mu \frac{\partial^{2}u}{\partial t\partial x}+\tau \left(x\right)$$

E(x) is the elastic modulus, μ is the viscosity coefficient, and τ(x) is the active stress. β(x) in (1) changes along the cell length and is given by:

(3) $$\beta \left(x\right)=\beta_{0}k_{s}{\left[\Psi_{1}+\left(1-\Psi_{1}\right)\frac{x-r}{f-r}\right]}^{-1}n_{f}$$

*β*_*0*_ is a constant, *k*_*s*_ is a coefficient for cell-substrate interaction, ψ_1_≥1 is the linear increase of dissociation rate towards the rear, *r* and *f* are the positions of the cell’s rear and front, respectively, and *n*_*f*_ is the density of free integrins.

Generally, integrin clustering is required to support cell locomotion as cell motility is regulated by varying ligand spatial presentation at the nanoscale level. As the dynamics of actin network formation is detailed in ref. [21], it is not represented here. The spatial distribution of actin network density is assumed time-independent with the following description derived in ref. [16], in which the dependency of the elastic modulus, E(x), to x can be expressed as:

(4) $$E\left(x\right)=E_{0}a\left(x\right)$$

Where *E*_*0*_ is a constant.

By approximate matching with the presented curve in the study of Gracheva *et al*. 2004 [16] the a(x) function is obtained as eq. 5:

(5) $$a\left(x\right)=2.381\times 10^{9}\left[\frac{2}{\pi }\arctan\left(700\left(x-x_{\mathrm{mid}}\right)\right)+2\right]\;mm$$

Finally, τ(x) can be calculated from:

(6) $$\tau \left(x\right)=-\tau_{0}\frac{K_{\mathrm{Reg}}^{+}}{K_{\mathrm{Reg}}^{-}}{\left[\Psi_{2}+\left(1-\Psi_{2}\right)\frac{x-r}{f-r}\right]}^{-1}{\left[\text{Reg}\right]}_{0}\frac{n_{b}^{\alpha }}{n_{b0}^{\alpha }+n_{b}^{\alpha }}\frac{K_{m}^{+}}{K_{m}^{-}}m_{f}a\left(x\right)$$

τ_0_ is a constant, *K*_Reg_^+^ and *K*_Reg_^−^ are the rate of activation and deactivation of bound myosin II, respectively,Ψ_2_≥1, [Reg]_0_ is the maximum level of regulatory protein, *n*_*b*_ is the density of integrins bound to the substrate, *n*_*b0*_ is its typical value, *α* is a degree of coupling between regulatory protein and integrins, *K*_*m*_^+^ and *K*_*m*_^−^ are the rate of myosin binding and decay of bound myosin, respectively, and *m*_*f*_ is the concentration of free myosins. Table 1 lists the cell parameters used in the calculations. It is further assumed that *E*_*0*_=0.42×10^-10^*N*/*mm*[22] and the viscosity is constant, μ=0.0002 *Ns*/*mm*^*2*^.

**Table 1:** **Cell parameters**[16]

	
*k*_*s*_	0.05
ψ_1_	3.33
ψ_2_	10
*k*_*m*_^+^/*k*_*m*_^−^	0.3
*k*_Reg_^+^[Re*g*]_0_/*k*_Reg_^−^	0.1
*α*	0.2
*β*_*0*_(*Ns*/*mm*^*3*^)	5×10^-6^
*n*_*f*_(*1*/*mm*)	5490
*n*_*b0*_ (*1*/*mm*)	12500
*m*_*f*_ (*1*/*mm*)	690
*τ*_*0*_ (*N*/*mm*)	4.2×10^-10^
*L*(*mm*)	0.05

**Figure 2:**
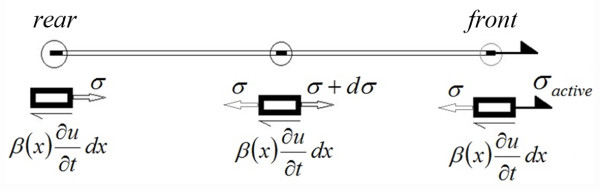
**Free**-**body diagram of the cell.**

According to the free-body diagram of the cell show in Figure 2, the equations of motion and the boundary condition can be presented as follows:

(7) $$\frac{\sigma_{\mathrm{active}}-\sigma }{du}=\beta \left(x\right)\frac{\partial u}{\partial t}leading\;edge$$

(8) $$\frac{\sigma }{du}=\beta \left(x\right)\frac{\partial u}{\partial t}trailing\;edge$$

There are two different boundary conditions in this model. First, the rear of the cell is fixed and active stress is applied to the front of the cell. During this time, the stress at the first point of the cell increases. When this stress exceeds a critical value (i.e. *σ*_C_^,^, the magnitude of the critical stress), the boundary condition changes. Next the front is fixed and the rear of the cell is released and starts to move toward the front. During the 2^nd^ course, the stress of the first point decreases and when it reaches to zero, the previous boundary condition becomes active. The procedure repeats during the cell movement.

In the first course, when the stress of the first point is still below the critical value, i.e. σ_1_<σ_c_, the B.C. is:

(9) $$\frac{\sigma_{\mathrm{active}}-\sigma }{dx}=\beta \left(x\right)\frac{\partial u}{\partial t}leading\;edge$$

(10) $$u_{1}=0\;trailing\;edge$$

In the second course, when σ_1_exceeds σ_c_, the B.C. changes to:

(11) $$u_{n}=0\;leading\;edge$$

(12) $$\frac{\sigma }{dx}=\beta \left(x\right)\frac{\partial u}{\partial t}trailing\;edge$$

In all the equations, *σ* is defined as in eq. 2. The generated stress by frontal applied load, σ_*active*_, is defined by the following equation:

(13) $$\sigma_{\mathrm{active}}=F_{\mathrm{active}}/S_{\mathrm{cell}}$$

In which *S*_*cell*_=30 μm^2^ and *F*_*active*_=1000 *nN*[23]. By setting the strain equation, *du*/*dx*, in eq. 2, and substituting eq. 2 in eq. 7, eq. 8 and eq. 9, and discretizing with finite difference method, the equation of motion becomes:

(14) $$\begin{array}{l} \left(\frac{M}{dx_{i}}\right)u_{i-1}^{j+1}-\left[\frac{\beta_{i}}{dt}+M\left(\frac{1}{dx_{i+1}}+\frac{1}{dx_{i}}\right)\right]u_{i}^{j+1}+\left(\frac{M}{dx_{i+1}}\right)x_{i+1}^{j+1}\\ =\left(-\frac{2E_{i-\frac{1}{2}}^{j}}{d_{x_{i}}\left(dx_{i}+dx_{i+1}\right)}+\frac{M}{d_{x_{i}}}\right)u_{i-1}^{j}+\left[\frac{2\left(\frac{E_{i+\frac{1}{2}}^{j}}{dx_{i+1}}+\frac{E_{i-\frac{1}{2}}^{j}}{dx_{i}}\right)}{dx_{i}+dx_{i+1}}-M\left(\frac{1}{dx_{i+1}}+\frac{1}{dx_{i}}\right)-\frac{\beta_{i}}{dt}\right]u_{i}^{j}\\ +\left(-\frac{2E_{i+\frac{1}{2}}^{j}}{dx_{i+1}\left(dx_{i}+dx_{i+1}\right)}+\frac{M}{dx_{i+1}}\right)u_{i+1}^{j+1}+\frac{\tau_{i-1}^{j}-\tau_{i+1}^{j}}{dx_{i}+dx_{i+1}} \end{array}$$

The boundary conditions of the front and rear of the cell in the steady state motion are obtained as follow:

Leading edge:

(15) $$\begin{array}{l} \left(\frac{\mu }{dtdx_{x-1}^{2}}\right)u_{n-1}^{j+1}+\left(-\frac{\mu }{dtdx_{n-1}^{2}}-\frac{\beta_{n}}{dt}\right)u_{n}^{j+1}\\ =\left(\frac{\mu }{dtdx_{n-1}^{2}}-\frac{E_{n}^{j}}{dx_{n-1}^{2}}\right)u_{n-1}^{j}+\left(\frac{E_{n}}{dx_{n-1}^{2}}-\frac{\mu }{dtdx_{n-1}^{2}}-\frac{\beta_{n}}{dt}\right)u_{n}^{j}+\frac{\tau_{n}-\sigma_{\mathrm{active}}}{dx_{n-1}} \end{array}$$

Trailing edge:

(16) $$\begin{array}{l} \left(-\frac{\mu }{dtdx_{1}^{2}}-\frac{\beta_{1}}{dt}\right)u_{1}^{j+1}+\left(\frac{\mu }{dtdx_{1}^{z}}\right)u_{2}^{j+1}\\ =\left(\frac{E_{1}}{dx_{1}^{2}}-\frac{\beta_{1}}{dt}-\frac{\mu }{dtdx_{1}^{2}}\right)u_{1}^{j}+\left(\frac{\mu }{dtdx_{1}^{2}}-\frac{E_{1}}{dx_{1}^{2}}\right)u_{2}^{j}-\frac{\tau_{1}}{dx_{1}} \end{array}$$

Where:

(17) $$M=\frac{2\mu }{\left(dx_{i}+dx_{i+1}\right)}$$

(18) $$E_{i+\frac{1}{2}}=\frac{E_{i+1}+E_{i}}{2}=\frac{E\left(x_{i+1}\right)+E\left(x_{i}\right)}{2}$$

(19) $$E_{i-\frac{1}{2}}=\frac{E_{i}+E_{i-1}}{2}=\frac{E\left(x_{i+1}\right)+E\left(x_{i}\right)}{2}$$

(20) $$dx_{i}=x_{i+1}-x_{i}$$

The superscripts *i* and *j* represent the cell node position and the time-step, respectively. In this work, the cell is divided into 100 parts with 101 nodes. The time step *dt* has to be less than the time constant of the viscoelastic model which is defined as the ratio of the viscosity to elasticity in Kelvin-Voight model. Here the minimum time constant is 0.00066 minutes. Therefore, *dt*=*0*.*0001* was chosen.

Considering the first set of boundary conditions (eq. 9 and eq. 10) and using eq. 2, we will have σ_1_ as:

(21) $$\sigma_{1}=E_{1}\frac{u_{2}}{dx_{1}}+\tau_{1}$$

When the second set of boundary conditions, eq. 11 and eq. 12, are applied, σ_1_ becomes:

(22) $$\sigma_{1}=E_{1}\frac{u_{2}^{j}-u_{1}^{j}}{dx}+\mu \frac{u_{1}^{j+1}-u_{1}^{j}}{dt}+\tau_{1}$$

Therefore, a general method is derived for obtaining*u*_*i*-*1*_, *u*_*i*_, and *u*_*i*+*1*_ in *j*+1^th^ timesteps from their values in the *j*^th^ timestep:

(23) $$Au^{j+1}=Au^{j}+C$$

Multiplying both sides by *A*^-*1*^results in:

(24) $$u^{j+1}=A^{-1}\left(Bu^{j}+C\right)$$

Therefore, by using a finite difference method, displacement of the cell nodes in each time step are calculated from the displacement in the previous time step. Since at *t*=*0 min*. the cell is stationary, *u*^*1*^=*0* is the initial boundary condition for eq. 24. The nodes displacements are calculated and x is updated. The matrices *A*, *B*, and *C* are regenerated accordingly. *t* is increased by one time step *dt* and the process continues until *t* reaches the final time.

## Calculation results

**Figure 3:**
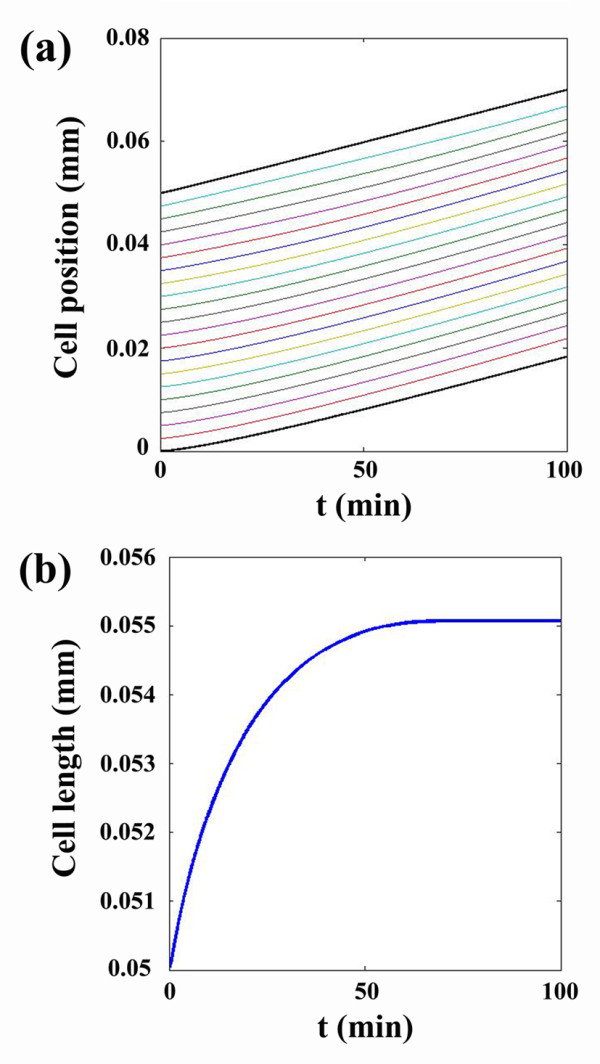
**(a)The cell position and(b)its length during the cell movement.**

In this study we first simulated 100 minutes of the steady movement of a cell. The results are shown in Figure 3 for the cell position and its length versus time. These results agree well with those of Gracheva *et al*. 2004 [16].

**Figure 4:**
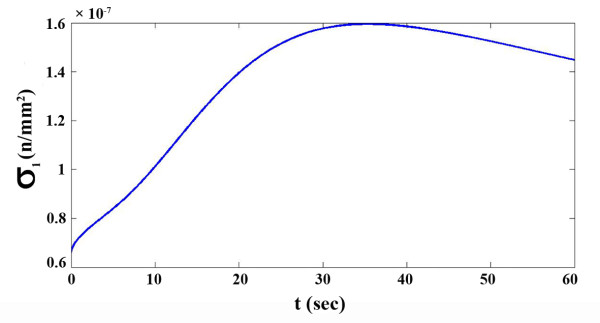
**Generated stress in the first point of the cell** (**rear**) **while the rear of the cell is fixed**, **and the active stress is applied to its front.**

The average cell speed was also in agreement with the experimental result of Lo *et al*. 2000 [24] estimating an average speed of 0.26±0.13 *μm*/*min*. Our model calculation resulted in 0.2 *μm*/*min*. This shows that the model is reasonably accurate and can be used for the ultimate model by applying a variable boundary condition, which is a function of the stress in the first node. Suppose that the rear part of the cell is fixed and the active stress is applied to its front part. The variation of the first node stress versus time is illustrated in Figure 4.

**Figure 5:**
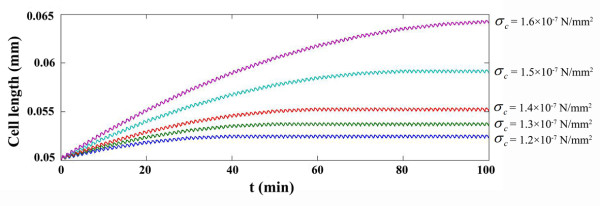
**The variation of the cell length during its movement for different values of σ**_**c**_**.**

**Figure 6:**
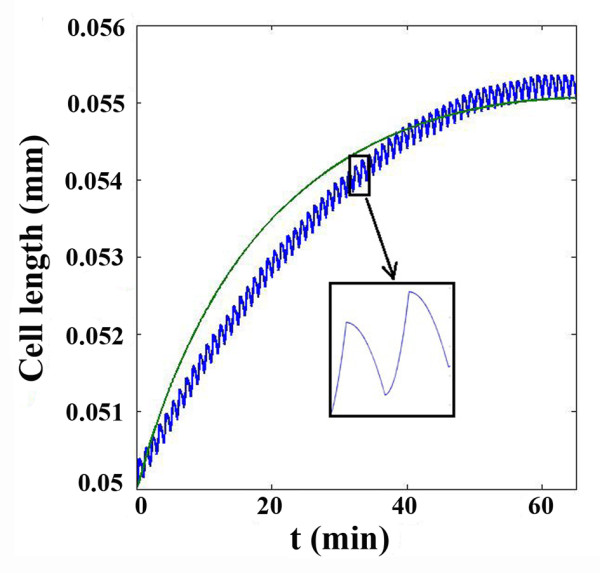
Cell length in steady and critical stress model.

As can be observed in Figure 4, *σ*_1_ has a maximum point at *σ*_1_ = 1.6 × 10^− 7^*N*/*mm*^2^. After this point,*σ*_1_ decreases slowly. This maximum value in Figure 4 was our first estimation for the critical stress parameter, *σ*_*c*_. For more accurate estimation, *σ*_*c*_ was decreased to 1.2 × 10^− 7^*N*/*mm*^2^ in five equal steps. In each step the results were compared with the steady results of the previous model. It was concluded that in state of *σ*_*c*_ = 1.4 × 10^− 7^*N*/*mm*^2^ the new model agrees with the results of the steady model. Figure 5 shows the cell length in different critical stress values. The similarity of the two models results can be observed in Figure 6 where the critical stress for the critical stress model has the appropriate magnitude of *σ*_*c*_ = 1.4 × 10^− 7^*N*/*mm*^2^.

**Figure 7:**
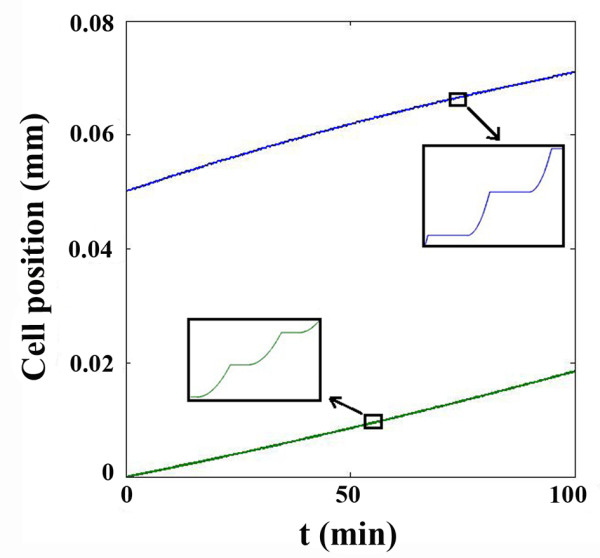
Calculated positions of the leading and trailing edges of the cell in critical stress model.

The average speed of the cell (which was previously estimated from Figure 3 in the steady stage), can be calculated from Figure 7 in the critical stress model, which gives 0.2 *μm*/*min*. This value is in agreement with the reported experimental data for the cell velocity [24].

For future work, we suggest the introduction of a self-regulatory mechanism that would act on the boundaries as the stress goes up. For instance, something that would change the dissociation rate as stress increases.

## Conclusion

Predicting and evaluating the cell movement, cell speed, and the generated stresses in the cell have been under consideration in recent decades. Mechanical models are gradually created to be able to give appropriate predictions of the cell motility processes. Based on experimental observations ameboid cell movement includes four steps of protrusion, adhesion to substrate, cell body movement and detachment of cell tail. In previous studies based on the viscoelastic continuum description of the cell motion, these steps have not been included in cell movement modeling and a steady movement was attributed to the cell [16,17]. Here, we promoted the previous models by changing the boundary conditions to more realistic assumptions. We analyzed the dynamics of the cell in our model and compared it with that of the previous models. In the new model the effect of adhesion to the substrate is considered through a cell-substrate interaction parameter along with the two-step boundary conditions that offers an acceptable survey of cell movement in different environments. The results of our model agree with the overall results of the steady model and provide additional information on the cell elongation and stress. The calculated cell velocity also agrees with the experimental value. The obtained results can assist nanoscale tissue engineering to achieve its main goal which is predicting cellular behaviour and interactions between cells and the environment by engineering the nanoscale presentation of biologically relevant molecular signals.

## Competing interests

The authors declare that they have no competing interests.

## Authors’ contributions

MM performed the initial modeling. FF, MM, DV and LT revised the results, modeling and discussions. All authors contributed to writing the manuscript, and agreed on its final contents. All authors read and approved the final manuscript.
